# TFA and EPA Productivities of *Nannochloropsis salina* Influenced by Temperature and Nitrate Stimuli in Turbidostatic Controlled Experiments

**DOI:** 10.3390/md8092526

**Published:** 2010-09-27

**Authors:** Maren Hoffmann, Kai Marxen, Rüdiger Schulz, Klaus Heinrich Vanselow

**Affiliations:** 1 Forschungs- und Technologiezentrum Westküste der Universität Kiel, Hafentörn 1, 25761 Büsum, Germany; E-Mails: hoffmann@ftz-west.uni-kiel.de (M.H.); marxen@ftz-west.uni-kiel.de (K.M.); 2 Botanisches Institut der Universität Kiel, Am Botanischen Garten 1–9, 24098 Kiel, Germany; E-Mail: rschulz@bot.uni-kiel.de (R.S.)

**Keywords:** microalgae, ratio unsaturated/saturated FA, growth rate, total fatty acids, eicosapentaenoic acid

## Abstract

The influence of different nitrate concentrations in combination with three cultivation temperatures on the total fatty acids (TFA) and eicosapentaenoic acid (EPA) content of *Nannochloropsis salina* was investigated. This was done by virtue of turbidostatic controlled cultures. This control mode enables the cultivation of microalgae under defined conditions and, therefore, the influence of single parameters on the fatty acid synthesis of *Nannochloropsis salina* can be investigated. Generally, growth rates decreased under low nitrate concentrations. This effect was reinforced when cells were exposed to lower temperatures (from 26 °C down to 17 °C). Considering the cellular TFA concentration, nitrate provoked an increase of TFA under nitrate limitation up to 70% of the biological dry mass (BDM). In contrast to this finding, the EPA content decreased under low nitrate concentrations. Nevertheless, both TFA and EPA contents increased under a low culture temperature (17 °C) compared to moderate temperatures of 21 °C and 26 °C. In terms of biotechnological production, the growth rate has to be taken into account. Therefore, for both TFA and EPA production, a temperature of 17 °C and a nitrate concentration of 1800 μmol L^−1^ afforded the highest productivities. Temperatures of 21 °C and 26 °C in combination with 1800 μmol L^−1^ nitrate showed slightly lower TFA and EPA productivities.

## 1. Introduction

Microalgae are capable of synthesizing marine drugs, such as antioxidants, antibiotics, vitamins, and toxins, which are of growing interest for the cosmetic, pharmacological, and food industry [1–3]. A further group of these marine drugs are fatty acids, a class of substances which can be synthesized and intracellularly accumulated in high amounts by microalgae [4–7].

Therefore, it has been widely discussed in recent years whether microalgae can be used for the production of biofuel and biodiesel [4,8–11]. Furthermore, the use of microalgae as a natural source of fatty acids for the aquaculture has also become the focus of industrial and scientific developments [12–14].

However, such approaches prefer two different kinds of fatty acids. Whereas for biodiesel production microalgae with high contents of saturated (SFA) and monounsaturated (MUFA) fatty acids (the main components of the total fatty acids (TFA)) are sought [4], the content of polyunsaturated fatty acids (PUFA) is crucial for the use of microalgae in aquaculture [15,16].

Actually, one of the most promising candidates of microalgae seems to be *Nannochloropsis salina*, because this microalga yields high amounts of TFA and/or PUFA [17,18]. Another important fact for the use of *Nannochloropsis salina* in aquaculture is that the main component of the PUFA in this alga is eicosapentaenoic acid (EPA; [19–21]), which is one of the favored fatty acids in the aquaculture [15].

Although scientific investigations have been published during the last two decades, considering the biotechnological potential of *Nannochloropsis salina* [17,21–23], a large scale production of this alga is not yet established.

Nevertheless, in order to exploit the potential of *Nannochloropsis salina* as a producer for marine drugs an adequate cultivation is required. Generally, the cultivation of microalgae seems to be easy, but reality often shows a different picture. Difficulties can result for example from the need of avoiding contamination, high energy consumption and cultivation conditions which are hard to control (e.g., temperature and light intensity).

The experimental setup presented here offers a detailed detection concerning the concentration of TFA and EPA under certain stress conditions. The investigated parameters are the cultivation temperature and nitrate concentration. This was done as it is difficult to control temperatures in outdoor cultivations (or at least requires high energy consumation and costly techniques) and nitrate is the highest concentrated and most expensive component in conventionally used f/2-medium for marine microalgae [24].

In contrast to other studies [17,21–23], a turbidostatic control mode was chosen. By virtue of this mode, defined conditions can be adjusted allowing the detection of the cellular influence of the investigated cultivation parameters and excluding possible changes of other parameters (e.g., pH, ratio of light intensity to cell number). The practical relevance of the investigated parameters and the cultivation mode enables an estimation of the productivities of *Nannochloropsis salina* in large scale productions influenced by nitrate and temperature changes.

## 2. Materials and Methods

### 2.1. Cultivation of the microorganisms

#### 2.1.1. Batch processes

*Nannochloropsis salina* (SAG 40.85) was obtained from the SAG culture collection in Göttingen (Germany). As inoculum for the turbidostatic processes, cells of *Nannochloropsis salina* were precultivated in batch cultures. After the end of these cultivations, the cells were transferred into the photobioreactor (see below).

The pre-cultures of *Nannochloropsis salina* were cultivated in 1L-bottles (Schott, Mainz, Germany) at a temperature of 22 °C and a photosynthetically active radiation (PAR) of 150 μmol photons m^−2^ s^−1^ (measured with a light sensor (US-SQS/LI, Walz, Effeltrich, Germany)). An 8-fold enriched f/2-concentration [24] in artificial sea water (32‰, Tropic Marin^®^, Tagis Tropical Marin, Dreieich, Germany) was employed as medium for the batch cultures. Via a gas flow controller (DK800N, Krohne, Germany) the CO_2_-concentration in the aeration was adjusted at 1%, whereby at the end of the batch cultivations the pH-values reached 8.5 ± 0.5. The aeration entered the reactor at the top and a silicon tube transferred the gas to the bottom, so that the aeration was also responsible for the mixing of the cell suspension.

After 10 to 14 days, the batch cultivations were stopped and the cells were transferred into the photobioreactors for the turbidostatic experiments. The optical density (OD) at the end of the batch cultures was measured in a spectrophotometer (U-1100, HITACHI, Tokyo, Japan). In order to receive convenient OD values, samples with OD values above 1 were diluted with medium. The optical density was then calculated by using the dilution volume. The OD reached values of 11 to 16 determined at a wavelength of 750 nm and biological dry masses between 2.2 mg L^−1^ and 3.1 mg L^−1^ were measured according to the method described below.

#### 2.1.2. Turbidostatic experiments

The transferred cells of *Nannochloropsis salina* were cultivated in a photobioreactor [25] under turbidostatic control (see below). The pH-value was adjusted to 8.1. Two glass tubes (SIMAX-glass, Kavalier, Savaza, Czech Republic, length 1.5 m, diameter 50 mm) of the photobioreactor enabled the illumination of the microalgae generated by two fluorescence tubes (TL58W/25, Osram, Munich, Germany) with continuous PAR of 200 μmol photons m^−2^ s^−1^.

Under turbidostatic control, the biomass concentration (and thus the cell density) in the reactor was kept constant (at 0.18 ± 0.02 mg L^−1^). By diluting the suspension of microorganisms with fresh medium in the reactor under the control of a feed-back loop, growth of the microorganisms was compensated, via an overflow outlet, the volume of the suspension (with constant biomass) which is equal to the added volume of fresh nutrient solution leaving the reactor. The fresh medium was taken from medium reservoirs, which provided different nitrogen concentrations (see below). For further information of the photobioreactor’s design and the turbidostatic control mode see Marxen *et al.* [25].

Nevertheless, it is important for the interpretation of results presented here, that due to the turbidostatic control, the ratios of cells and PAR irradiation were equal in all experiments and therefore the PAR irradiance could be excluded for the determined effects.

### 2.2. Experimental nitrate and temperature setup

For the turbidostatic cultures of *Nannochloropsis salina,* a doubled concentration of f/2-medium [24] was applied. In order to investigate the influence of nitrogen, different concentrations of nitrate were tested. The highest nitrate concentration of 1800 μmol L^−1^ NO_3_ ^−^ corresponded to the normal nitrate concentration according to the reference mentioned above. Therefore, this nitrate concentration was applied to simulate “unstressed” growth of *Nannochloropsis salina*. Nitrate concentrations of 600 and 300 μmol L^−1^ were employed in order to simulate moderate nitrate stress, whereas nitrate concentrations of 150 and 75 μmol L^−1^ NO_3_ ^−^ should provide high stress conditions.

The different nitrate concentrations were provided in the medium reservoirs from which the turbidostatic control mode pumped fresh medium into the reactor to keep the optical density of the cell suspension constant.

Additionally, the experimental nitrate setup was tested at two temperatures (21 °C and 26 °C). In order to simulate higher stress conditions, the lowest nitrogen concentration of 75 μmol L^−1^ was additionally tested at a temperature of 17 °C. As reference for this temperature, a full nitrate concentration of 1800 μmol L^−1^ NO_3_ ^−^ was also applied.

All experiments were conducted for 340 h.

### 2.3. Determination of growth rate (μ) and biological dry mass (BDM)

The growth rates μ of the microorganisms were calculated during the turbidostatic processes as follows [26]:

(1) $$\mu =\frac{\Delta V_{R}}{V_{L}\times \Delta t}$$

In this equation, V_L_ was the liquid reactor volume, ΔV_R_ was the difference of the added volume of fresh medium (due to the turbidostatic process) and Δt was the considered time interval. Every 24 h the growth rates were calculated.

A glass microfibre filter (Ø 25 mm, Whatman, Brentfort, UK) was combusted and weighed. Afterwards, a 2 mL sample from the reactor vessel was taken and filtered. After heating (104 °C) for 24 h the filter was weighed again. The resulting difference was used for calculating the biological dry mass. Samples for the determination of BDM were taken in triplicate. Average and standard deviation were calculated.

### 2.4. Fatty acids determination

For the determination of both the fatty acid composition and concentrations the protocol of Garcés and Mancha [27] was applied.

Sample volumes of 45 mL were taken from the reactor, freeze dried and mixed with 2 mL of a chemical solution containing four different components: methanol, toluol, concentrated sulphuric acid, and 2,2-dimethoxypropane (39:10:2:1 (v:v:v:v)). Internal fatty acid standards (nonadecanoic acid (C19:0, 1000 ppm) and tricosan acid (C23:0, 500 ppm)) and 1.5 mL of pure hexan were added to the prepared sample. After overlaying the samples with pure nitrogen, the samples were treated in an ultrasonic bath (RK100H, Bandelin, Berlin, Germany) for 5 min. Afterwards, the samples were stored in a thermo block (2050-1CESUP, Barnstead/Lab-Line, Melrose Park, IL, USA) at 80 °C for 2 h. After cooling down to room temperature, the upper phase of the samples were taken and evaporated with pure nitrogen. Immediately prior to the analysis, 0.5 mL of pure hexane was added to the prepared sample.

Subsequently, the analysis was performed using a gas chromatograph (GC-14B, Shimadzu, Kyoto, Japan). The temperature of the injector and detector were adjusted at 250 °C and 280 °C respectively. The separation of single fatty acids was carried out with a capillary column (FS-CW 20M-CB, 30 m × 0.25 mm × 0.31 μm, CS-Chromatographie; Langerwehe, Germany). Helium was used as carrier gas at a flow rate of 1.3 mL min^−1^. The temperature program was as follows: 80 °C for 0.5 min, 25 °C min^−1^ up to 200 °C, and 3 °C min^−1^ up to 230 °C for 17 min.

In this study the fatty acids were divided into two groups: total fatty acid content (TFA) which contains the complete fatty acid concentration, and the polyunsaturated fatty acid eicosapentaenoic acid (EPA) was presented separately.

Samples for the determination of fatty acid concentrations and compositions were taken in duplicate. In case of the experiment conducted at 21 °C samples were taken every 24 h, whereas, at the two other experiments samples were taken only at the end, when steady state conditions of the microalgae were reached (see below).

In order to eliminate slight differences of the BDM-values, fatty acid concentrations were normalized to measured BDM-values. Average and standard deviation were calculated from the duplicates.

### 2.5. Fatty acid productivity (P_x_)

For the calculation of productivity, the normalized concentrations of the fatty acid measurements were implemented in the following equation:

(2) $$P_{x}=\mu \times c_{x}$$

In Equation 2, P_x_ represents the productivity and x was used as an index for the different fatty acid groups, μ is the growth rate (see above) and c_x_ is the considered concentration of the product (TFA or EPA).

## 3. Results

### 3.1. Biological dry mass (BDM) and growth rate (μ)

**Figure 1:**
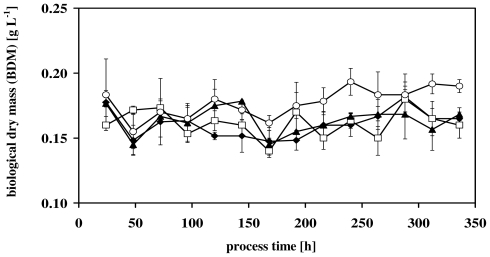
Biological dry mass (BDM) of some representative turbidostatic experiments at 21 °C. Different nitrate concentrations (μmol NO_3_ ^−^ L^−1^): (♦) 75; (□) 150; (▴) 300; (○) 600.

In Figure 1 some representative time courses of the measured BDM-values during the turbidostatic experiments at 21 °C are shown. There are no trends with respect to the different nitrate concentrations detectable. This holds also for the two other culture temperatures of 26 °C and 17 °C.

Table 1 summarizes all measured BDM-values for all experiments. It is obvious that none of the investigated parameters (nitrate concentration and cultivation temperature) led to a significant influence. The constancy of BDM in Figure 1 and the results presented in Table 1 demonstrates the reliability of the turbidostatic control.

**Figure 2:**
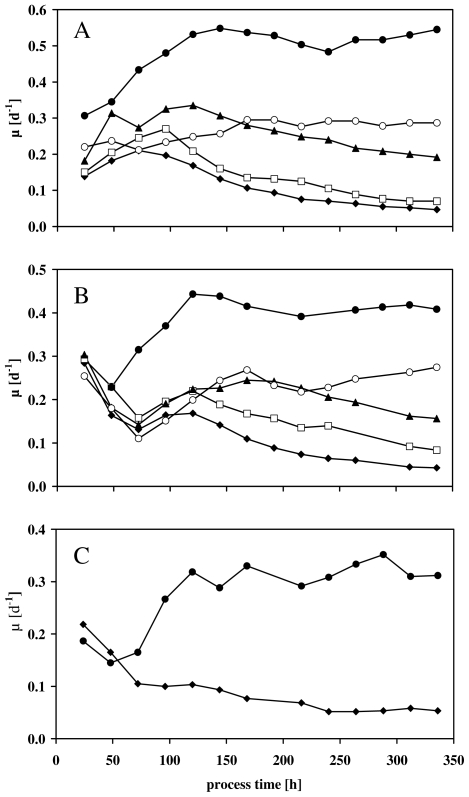
Growth rate μ of the turbidostatic experiments with different nitrate concentrations at (**A**) 26 °C; (**B**) 21 °C and (**C**) 17 °C. Nitrate concentrations (μmol NO_3_ ^−^ L^−1^): (♦) 75; (□) 150; (▴) 300; (○) 600; (●) 1800.

By means of Equation 2 the time courses of the calculated growth rates showed a complete different picture in Figure 2. At the beginning of the experiments μ started in a range between 0.3 and 0.15 d^−1^ (Figure 2). After about 150 h of the process time, μ showed a more or less pronounced adaptation phase for all experiments after the transfer of cells from the batch cultures into the turbidostatic processes (Figure 2A–C).

Nevertheless, it seemed clear that the temperature influenced μ for the experiments with the highest nitrate concentration of 1800 μmol NO_3_ ^−^ L^−1^ (Figure 2 A–C). After 340 h of the experiments μ fell from 0.55 d^−1^, at the highest temperature of 26 °C (Figure 2A), down to 0.3 d^−1^, at the lowest temperature of 17 °C (Figure 2C).

More pronounced than the temperature was the influence of nitrate limitation on μ. Independent of the applied temperature (26 °C or 21 °C), the growth rates of nitrate limited experiments fell according to the employed nitrate concentrations. Whereas moderate nitrate concentrations of 600 and 300 μmol NO_3_ ^−^ L^−1^ resulted in a μ-range of between 0.3 and 0.2 d^−1^ (Figure 2A, B); the lowest nitrate concentrations of 150 and 75 μmol NO_3_ ^−^ L^−1^ forced μ-values lower than 0.1 d^−1^ at the end of the experiments (Figure 2A–C).

### 3.2. Fatty acids

**Figure 3:**
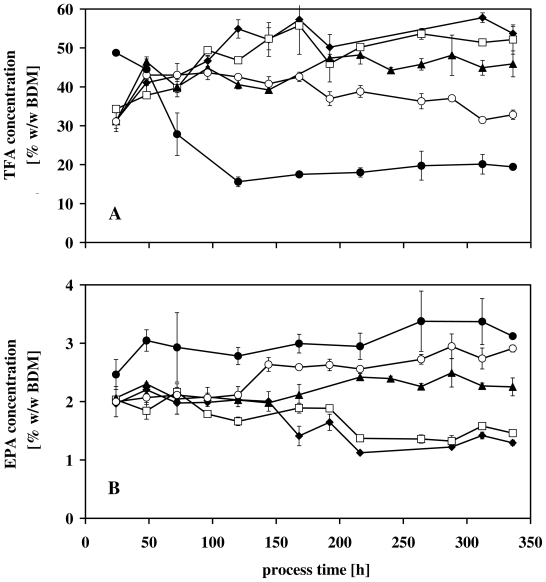
Representative time courses of the total fatty acid concentration (TFA) and eicosapentaenoic acid (EPA) at 21°C and different nitrate concentrations. (**A**) TFA concentration [% w/w BDM]; (**B**) EPA concentration [% w/w BDM] Nitrate concentrations [μmol NO_3_ ^−^ L^−1^]: (♦) 75; (□) 150; (▴) 300; (○) 600; ● 1800.

In Figure 3 representative time courses of TFA and EPA at a cultivation temperature of 21 °C and different nitrate concentrations were depicted. It was obvious that, after the transfer into the turbidostatic controlled photobioreactor, cells adapted to the new cultivation conditions during the first 75–125 h of the process time. Afterwards different developments of the TFA and EPA concentrations were observable.

The TFA concentration of the cells provided with 1800 μmol L^−1^ NO_3_ ^−^ decreased from 50% w/w BDM down to 20% w/w BDM (Figure 3A), whereas the EPA content increased to 3% w/w BDM (Figure 3B).

Figure 3 revealed another important fact, which is necessary for the understanding of the experiments and the interpretation of the data. At the end of the experiments (t > 300 h of the process time), TFA and EPA concentrations reached steady state values. Therefore, only these values could be taken into consideration for the comparison of both nitrate influenced synthesis of TFA and EPA and the calculated productivities by means of Equation 2.

**Figure 4:**
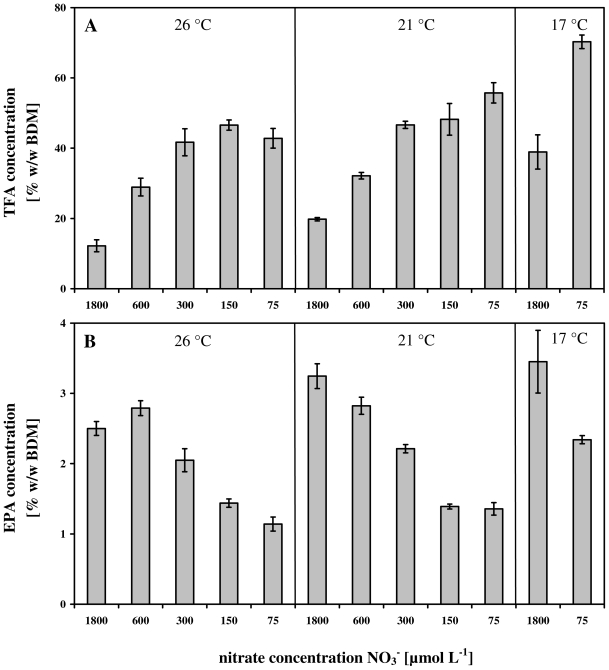
Results of the total fatty acid concentration (TFA) and eicosapentaenoic acid (EPA) at 26 °C, 21 °C and 17 °C and different nitrate concentrations at the end of the experiments: (**A**) TFA concentration [% w/w BDM]; (**B**) EPA concentration [% w/w BDM].

In Figure 4, the strong influence of different nitrate levels in combination with different cultivation temperatures (see Materials and Methods section) on final cellular concentrations of TFA and EPA in *Nannochloropsis salina* was depicted.

The TFA concentrations tended towards nitrate limitation and low cultivation temperatures. For example, a temperature of 17 °C and the lowest nitrate concentration of 75 μmol L^−1^ induced a TFA content of 70% w/w BDM, which is nearly 6-times higher than final TFA concentrations (12% w/w BDM) reached with full nitrate supplement and highest temperature of 26 °C (Figure 4A).

A different trend was observable considering the EPA concentrations. Similar to the TFA contents, low temperatures again provoked an increase of the EPA content (Figure 4B). However, in contrast to the nitrate induced effect mentioned above, *Nannochloropsis salina* reacted to low nitrate supply by a down regulation of the cellular EPA concentrations (Figure 4B). For example the cellular EPA content at 21 °C was 2.5-times lower at 75 μmol L^−1^ NO_3_ ^−^ (1.3% w/w BDM) compared to 1800 μmol L^−1^ NO_3_ ^−^ (3.3% w/w BDM).

Nevertheless, it has to be mentioned that in both cases the increase of TFA and the decrease of EPA seemed to correlate with the provided nitrate concentration (Figure 4A, B).

To investigate the ratio of unsaturated to saturated fatty acids, we focused on the content of fatty acids in *Nannochloropsis salina*. The major components were C14:0, C16:0, C16:1, C18:1n9, C18:2n6, 18:3n6, C20:4n6 and C20:5n3 and their total amount was approximately 95% w/w TFA (Table 2). Other fatty acids (saturated and unsaturated) were minor components with approximately 5% w/w TFA.

The fatty acids C16:0 and C16:1 represent about 70% to 80% of TFA and thus, they define the ratio unsaturated/saturated in a crucial way. With declining nitrate concentrations C16:0 showed a marginal increasing trend, whereas the content of C16:1 remains nearly constant. Solely, the fatty acid C18:1n9 in the group of unsaturated fatty acids, showed an increased concentration with decreasing nitrate concentration.

The environmental factors nitrate and temperature have only a little influence on the ratio of unsaturated to saturated fatty acids in *Nannochloropsis salina* (Table 2). The overall average ratio of all experiments was 1.16 (±0.3). The highest ratio with 1.92 is detectable in the non-N-depleted culture at 17 °C and the lowest ratio with 0.87 was calculated in the culture with 300 μmol NO_3_ ^−^ at 26 °C.

### 3.3. Productivities

**Figure 5:**
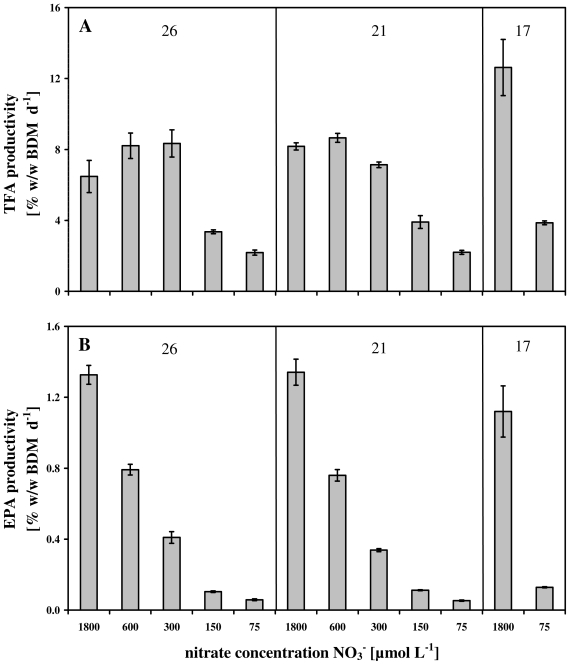
Productivity of the total fatty acid (TFA) and eicosapentaenoic acid (EPA) at 26 °C, 21 °C and 17 °C and different nitrate concentrations at the end of the experiments. (**A**) TFA productivity [% w/w BDM d^−1^]; (**B**) EPA productivity [% w/w BDM d^−1^].

By means of Equation 2, the calculated productivities of TFA and EPA at the end of the experiments were depicted in Figure 5. It has to be taken into account that Equation 2 summarizes the effects of nitrate concentrations and culture temperature on both fatty acid concentrations and growth rates. Therefore, the positive influence of nitrate limitation on the TFA concentration in *Nannochloropsis salina* (Figures 3A and 4A) did not necessarily lead to an increased TFA productivity since μ strongly decreased under nitrate limitation (Figure 2).

For example, the cellular TFA content reached a maximum of 70% w/w BDM at a temperature of 17 °C and the lowest nitrate concentration of 75 μmol L^−1^ NO_3_ ^−^ (Figure 4A), but the low growth rate at this point of 0.55 d^−1^ (Table 1) resulted in productivity of 3.5% w/w BDM d^−1^. At the same temperature, when cells were exposed to a nitrate concentration of 1800 μmol L^−1^, the TFA content was only half (37% w/w BDM) of the concentration mentioned above, but the growth rate of 0.32 d^−1^ (Table 1) enhanced the TFA productivity up to 13% w/w BDM d^−1^.

From full nitrate concentrations of 1800 μmol L^−1^ down to moderate nitrate limitation of 300 μmol L^−1^, the TFA productivities showed nearly same values of 6–8% w/w BDM d^−1^ at 21 °C and 26 °C (Figure 5A), which are 2.2- to 1.6-times lower than the maximum TFA productivity of 13% w/w BDM d^−1^.

This finding was even more pronounced concerning the EPA productivity (Figure 5B). At all three temperatures nitrate concentrations of 1800 μmol L^−1^ induced the highest productivities. Although at 17 °C EPA productivity of 1.3% w/w BDM d^−1^ was below the values of 21 °C and 26 °C, which are nearly identical (1.7% w/w BDM d^−1^), these three productivities were much higher than the other productivities provoked by nitrate limitations (Figure 5B).

Therefore, the summarized observation is that in the experiments presented in this study nitrate limitation did not lead to a remarkable increase of TFA or EPA productivity due to dependence on μ (Equation 2).

## 4. Discussion

### 4.1. Biological dry mass (BDM) and growth rate (μ)

Since changes of culture conditions could influence both the fatty acid concentrations and compositions [4,12,28], defined experimental conditions have to be adjusted and controlled for experiments investigating the influence of single parameters on fatty acid synthesis. This was done by virtue of both instrumental setup of the used photobioreactor [25] and turbidostatic control mode [25,29]. Therefore, changes of controlled parameters, for example, pH-value, the PAR-intensity per cell ratio could be excluded as stimuli for the determined effects. As mentioned above the constancy of the BDM values indicated the reliability of the turbidostatic control mode and ensured that the measured effects at the end of the experiments could be assigned to the employed different nitrate concentrations and temperatures.

Considering the points mentioned above, the constant growth rates at the end of the experiments (Figure 1) revealed some remarkable points. After transferring the cells from the batch cultures into the turbidostatic processes μ decreased and recovered within the first 150 h of the process time. This could be interpreted as an adaptation phase in which the cells adapt to new PAR intensity per cell ratios; a cellular response, which seems to be typical for phototrophic microorganisms [30,31].

The constant growth rates at the end of the experiments (Figure 1) were smaller compared to growth rates presented in other studies [32–34]. But it has to be taken into account that different process strategies and experimental designs provoke different growth rates of phototrophic growing microorganisms. Therefore, it is hardly possible for a direct comparison of the growth rates, but changes of the growth rate induced by different temperatures [32,33] and nitrogen concentrations [33] were similar to the findings presented in this study (Figure 1).

Furthermore, the influence of nitrate limitation on growth rates seems to be more pronounced than under temperature changes (Figure 2 and [33]). The calculated μ-values (Figure 2) implied the strong dependence of cell replication on the provided nitrate concentration. This might have been caused by the decreased activity of proteins, for example, Rubisco [35], and reduced synthesis of proteins and chlorophyll *a* under nitrate limitation [36]. This loss of chlorophyll *a* led to a reduced efficiency of energy collection which is required for cell replication [36].

Nevertheless, considering the results presented in Figures 2 and 4, nitrogen starvation led to an enhanced synthesis of the TFA content, which seems to be coupled with the decline of the growth rates.

### 4.2. Fatty acids and temperature

Both, cellular TFA and EPA concentrations are known to be parameters strongly influenced by temperatures. Temperatures below the optimal cultivation temperature seem to lead to a decreased synthesis of saturated fatty acids and increased concentrations of unsaturated fatty acids [4,21,37,38].

However, in this study the TFA content, which predominantly represented the saturated and monounsaturated fatty acids [20], increased with decreasing temperatures (Figure 4A). This could have been an effect provoked by a temperature induced limited uptake of nitrogen. Both, nitrate uptake and the reduction of the absorbed nitrate to ammonia are known to be temperature sensitive [39]. These processes could lead to a nitrogen induced loss of chlorophyll *a* [35], which results in an overreduced state of the electron transport chain (ETC). Nevertheless, the cells counteract this problem by an enhanced accumulation of short-chain saturated and monounsaturated fatty acid containing triacyglycerols (TAGs) [20], a reaction which also occurs under purely nitrate limitation (described below) and which is a possible reason for the enhanced TFA concentrations (Figure 4A).

Low temperatures negatively affect the membrane fluidity and cells counteracted by increased synthesis of polyunsaturated fatty acids and therefore EPA [40,41]. Furthermore, an enhanced EPA content in glycerol lipids of the thylakoid membranes protects the photosynthetic apparatus against low temperatures [42]. This is corroborated by the fact, that photosystem II is the most temperature sensitive component of the photosynthetic apparatus [43,44]. Therefore, the enhanced EPA concentration seemed to be a protection mechanism of *Nannochloropsis salina* against low temperature conditions.

Nevertheless, the influence of temperature on *Nannochloropsis salina* seemed to be complex and still difficult to explain in detail [45]. For example, the negative influence of temperature on TFA content described by Boussiba *et al.* [22] is contrary to the findings presented in this study or in Sukenik *et al.* [19] and Hu and Gao [21]. Further investigations are required for a more detailed insight into temperature induced physiological adaptions in *Nannochloropsis salina*.

### 4.3. Fatty acids and nitrate

*Nannochloropsis salina* counteracts nitrate limitation by the enhanced synthesis of the TFA concentration. A 5.8-fold higher concentration up to 70% of the BDM at 17 °C and lowest nitrate concentration of 75 μmol L^−1^, compared to an unstressed culture at 26 °C (with only 12% of the BDM), were observed (Figure 4A, Table 1). These findings were consistent with the results of Sukenik *et al.* [20] and Hu and Gao [21], although both studies employed different cultivation processes and extraction methods for the measurement of TFA contents than the ones presented here.

Generally, under nitrate limitations microalgae favor the synthesis of neutral lipids more than of polar lipids [46,47]. These neutral lipids are located in lipid bodies in the cytoplasm of the cells. They serve for the maintenance of cells under nitrate limitation [32,48].

Another important aspect of the high TFA content could be a protection of the photosynthetic apparatus of *Nannochloropsis salina*. Nitrate limitation might be responsible for the excess accumulation of electrons in the electron transport chain (ETC) generated by the light driven photosystems. This accumulation induces an overproduction of reactive oxygen species (ROS) [49,50], which negatively affect both photosynthesis and membrane lipids [51].

The synthesis of a C18 fatty acid requires approximately 24 molecules of NADPH, which were generated by the ETC, which is twice as much as for the synthesis of, for example, carbohydrates and protein molecules [4]. Therefore, the fatty acid synthesis leads to a relaxation of an overreduced ETC [4,52,53], which may occur under nitrate limitations.

However, in case of the EPA contents, nitrate deficiencies led to a completely different picture (Figure 4). EPA belongs to a group of fatty acids which are part of the glycerol lipids and serve as structural components within the cells [17].

These components are part of the chloroplasts and under nutritional limitations, such as nitrogen, cells are unable to resynthesize them and/or even keep the concentration of these components constant [54].

However, with adequate nutrition, cells are capable to synthesize high amounts of energy rich PUFAs, such as EPA [54]. Sukenik *et al.* [19,20] also described this trend, but Hu and Gao [21] published results which showed an increase of the EPA concentration of *Nannochloropsis salina* in batch cultures supplied with both high and low nitrate concentrations. It has to be taken into account that during discontinuous cultures the light per cell ratio changes due to cell growth. This might be a reason for the contrary results of Hu and Gao [21] and Sukenik *et al.* [19,20]. In this study, the turbidostatic process excludes the influence of changing light per cell ratios and the measured increased EPA content of *Nannochloropsis salina* could be explained by the employed nitrate concentrations.

The ability of *Nannochloropsis salina* to synthesize and accumulate high concentrations of EPA could be based on their contribution to some cell components. EPA is essential for the structure and stability of cell membranes [32]. Due to the high content of EPA in these membrane lipids [55,56], the synthesis of EPA delivers the basis for cell components which are the location of the photosynthetic apparatus [32,57] and, therefore, EPA is one essential substance which enables *Nannochloropsis salina* to generate energy by photosynthesis.

The utilization of bio-oil as a renewable energy resource and chemical feedstock requires certain properties; thereof, the ratio of unsaturated to saturated fatty acids is one of the most relevant. Dimian *et al.* [58] describes the heterogeneous catalysis for the production of biodiesel. For this process, a low ratio of unsaturated to saturated fatty acids in the feedstock is preferred because unsaturation slows down the reaction of the catalysis by steric and physical effects. The constancy of the ratios obtained at the end of the experiments and the comparison of the low overall average ratio (~1.16) of *Nannochloropsis salina* (Table 2) with the ratios of e.g., 9 for rapeseed, 5.25 for soya oil, or 5.8 for peanut oil (data from [58]) indicate the potential of this alga for the production of biodiesel.

### 4.4. Productivities

Two of the most crucial problems of using microalgae as source for commercial applications are the cellular concentrations and the productivity of the desired microalgal components.

In this case it has been clearly demonstrated, that nitrate and temperature can be used as stimuli for *Nannochloropsis salina* to synthesize and accumulate high TFA and EPA concentrations (Figures 3 and 4). In terms of commercial implementations of these results, Figure 5 summarizes by means of Equation 2 the impact of both parameters on the productivity. In both cases, TFA and EPA productivities, nitrate limitations led to a dramatic decrease of the growth rate (Figure 2) and therefore to low productivities (Figure 5). In contrast to this, different cultivation temperatures in combination with an appropriate nitrate concentration (e.g., 1800 μmol NO_3_ ^−^ L^−1^) stimulated TFA and EPA synthesis (Figure 4). For lower unsaturated fatty acids Ota *et al.* [59] described an increased productivity after nitrate depletion. This could not be validated in this study. The constancy of C16:1, the increase of C18:1n9 in Table 2 and the strong decrease of μ (shown in Table 1) result in a lowering of the productivities (Equation 2).

The resulting productivities of TFA and EPA (Figure 5) could be used for the estimation of outdoor cultivations (e.g., in green houses) which are often not temperature controlled. At moderate environmental temperatures, commercial productions could be shifted towards a TFA production, whereas at warm temperatures EPA production could be focused on the production process.

However, it has to be mentioned that the productivities presented in this study did not result from optimized cultivation protocols, and due to the low BDM values (Figure 1), the calculated productivities of TFA and EPA were below the values presented in other studies [18,21,33,60]. It has to be taken into account that these productivities were reached during batch processes. Due to the diversity of factors which were involved (e.g., continuously or discontinuously culture, light per cell ratio, pH value, temperature), no direct comparison of the productivities presented here and by others published recently [18,21,60] could be made. However, basic trends are comparable and verify the results presented in this study.

## 5. Conclusions

The experimental setup presented in this study showed that *Nannochloropsis salina* is able to synthesize high cellular concentrations of TFA and EPA and this ability could be stimulated via nitrate limitation and/or cultivation temperatures. However, in terms of a biotechnological implementation, both parameters negatively influenced the growth rate of *Nannochloropsis salina* and therefore lowered the TFA and EPA productivities during the turbidostatic experiments.

Nevertheless, the low ratios of the sums of unsaturated to saturated fatty acids in Table 2 indicate the suitability of *Nannochloropsis salina* as a potential source for the production of biodiesel.

## Figures and Tables

**Table 1:** Data represents the steady-state conditions at the end of the experiments.

Temperature [°C]	Nitrate concentration [μmol NO_3_^−^ L^−1^]	BDM [g L^−1^]	μ [d^−1^]	TFA [% w/w BDM]	EPA [% w/w BDM]
**26**	1800	0.22 ± 0.02	0.53	12 ± 2	2.5 ± 0.1
	600	0.17 ± 0.01	0.28	29 ± 3	2.8 ± 0.1
	300	0.16 ± 0.01	0.20	42 ± 4	2.1 ± 0.2
	150	0.16 ± 0.01	0.07	47 ± 2	1.4 ± 0.1
	75	0.16 ± 0.01	0.05	43 ± 3	1.1 ± 0.1

**21**	1800	0.21 ± 0.03	0.41	20 ± 1	3.3 ± 0.2
	600	0.18 ± 0.01	0.27	32 ± 1	2.8 ± 0.1
	300	0.16 ± 0.01	0.15	47 ± 1	2.2 ± 0.1
	150	0.16 ± 0.01	0.08	48 ± 5	1.4 ± 0.1
	75	0.16 ± 0.01	0.04	56 ± 3	1.4 ± 0.1

**17**	1800	0.18 ± 0.02	0.32	39 ± 5	3.5 ± 0.5
	75	0.18 ± 0.03	0.06	70 ± 2	2.3 ± 0.1

**Table 2:** Percentage values of all detected fatty acids in [% w/w TFA] of *Nannochloropsis salina*. Samples were taken at the end of the experiments. Values with standard deviations. Sat = Saturated, Unsat = Unsaturated.

Fatty acid group	Cultivation conditions: temperature [°C] and nitrate concentration [μmol L^−1^]											
	17		21					26				
	1800	75	1800	600	300	150	75	1800	600	300	150	75
C14:0	3.97 ±0.13	2.94 ±0.10	4.79 ±0.12	3.53 ±0.14	3.46 ±0.10	3.01 ±0.12	3.12 ±0.12	7.60 ±0.51	4.72 ±0.20	3.85 ±0.07	3.18 ±0.39	3.03 ±0.26
C16:0	37.51 ±0.69	38.06 ±0.56	34.71 ±0.58	42.18 ±0.66	43.35 ±0.54	43.18 ±0.77	43.06 ±0.37	31.26 ±1.24	42.20 ±0.53	46.82 ±0.81	46.48 ±0.96	46.11 ±1.24
C16:1	36.80 ±0.89	37.74 ±0.16	33.77 ±0.51	35.17 ±0.58	35.79 ±0.51	34.89 ±0.60	35.21 ±0.51	32.12 ±1.33	31.57 ±0.36	31.51 ±0.82	32.29 ±0.25	32.14 ±0.69
C18:1n9	2.85 ±0.05	11.18 ±0.05	1.48 ±0.04	2.60 ±0.06	4.35 ±0.11	7.62 ±0.14	9.60 ±0.16	1.10 ±0.49	2.55 ±0.06	4.39 ±0.28	7.12 ±0.65	8.09 ±0.50
C18:2n6	0.48 ±0.01	0.47 ±0.02	0.52 ±0.01	0.53 ±0.01	0.52 ±0.02	0.4 ±0.01	0.31 ±0.01	0.81 ±0.06	0.59 ±0.02	0.64 ±0.01	0.59 ±0.05	0.51 ±0.03
C18:3n6	0.91 ±0.03	0.89 ±0.09	1.05 ±0.19	1.45 ±0.38	1.60 ±0.14	1.36 ±0.26	1.03 ±0.20	0.93 ±0.25	1.46 ±0.45	1.33 ±0.27	1.10 ±0.31	1.35 ±0.23
C20:4n6	1.44 ±0.07	0.82 ±0.04	2.73 ±0.08	1.78 ±0.09	1.23 ±0.08	0.98 ±0.04	0.86 ±0.04	2.78 ±0.02	2.25 ±0.15	1.46 ±0.08	1.37 ±0.12	1.27 ±0.09
C20:5n3 (EPA)	8.65 ±0.54	3.34 ±0.10	16.32 ±0.46	8.77 ±0.30	4.76 ±0.27	2.91 ±0.14	2.43 ±0.11	17.39 ±2.02	10.05 ±0.55	4.95 ±0.15	3.21 ±0.06	2.70 ±0.15
Others (Sat)	3.75 ±0.42	2.8 ±0.07	2.93 ±0.21	2.35 ±0.08	2.82 ±0.24	2.89 ±0.17	2.75 ±0.16	4.17 ±1.44	2.68 ±0.43	2.79 ±0.30	2.61 ±0.24	3.33 ±1.73
Others (Unsat)	3.57 ±2.34	1.73 ±0.53	1.70 ±0.14	1.65 ±0.23	2.13 ±0.33	2.76 ±1.03	2.63 ±0.19	1.85 ±0.94	1.94 ±0.60	2.26 ±0.57	2.07 ±0.66	1.47 ±0.78

∑Sat	45.13 ±0.70	43.80 ±0.73	42.43 ±0.06	48.06 ±0.54	49.63 ±0.22	49.08 ±0.90	48.92 ±0.65	43.03 ±0.55	49.60 ±0.91	53.46 ±0.87	52.26 ±0.34	52.47 ±0.71
∑Unsat	54.87 ±0.70	56.18 ±0.70	57.57 ±0.58	51.94 ±0.61	50.37 ±0.41	50.92 ±0.64	51.08 ±0.24	56.97 ±0.83	50.40 ±0.49	46.54 ±0.66	47.74 ±0.64	47.53 ±1.11

∑Unsat/∑Sat	1.92 ±0.12	1.39 ±0.06	1.36 ±0.03	1.08 ±0.02	1.01 ±0.01	1.04 ±0.03	1.04 ±0.01	1.32 ±0.05	1.02 ±0.02	0.87 ±0.03	0.91 ±0.01	0.91 ±0.03
